# Potent Antiarthritic Properties of Phloretin in Murine Collagen-Induced Arthritis

**DOI:** 10.1155/2016/9831263

**Published:** 2016-12-04

**Authors:** Shun-Ping Wang, Shih-Chao Lin, Shiming Li, Ya-Hsuan Chao, Guang-Yuh Hwang, Chi-Chen Lin

**Affiliations:** ^1^Department of Life Science, Tunghai University, Taichung, Taiwan; ^2^Department of Orthopaedics, Taichung Veterans General Hospital, Taichung, Taiwan; ^3^Ph.D. Program in Medical Biotechnology, National Chung Hsing University, Taichung, Taiwan; ^4^SRI International, Harrisonburg, VA, USA; ^5^Hubei Key Laboratory of Economic Forest Germplasm Improvement and Resources Comprehensive Utilization and Hubei Collaborative Innovation Center for the Characteristic Resources Exploitation of Dabie Mountains, Huanggang Normal University, Huanggang, Hubei, China; ^6^Institute of Biomedical Science, National Chung Hsing University, Taichung, Taiwan; ^7^Department of Biotechnology, Asia University, Taichung, Taiwan; ^8^Department of Medical Research, China Medical University Hospital, Taichung, Taiwan

## Abstract

In the exploration of potential therapeutic agents for rheumatoid arthritis (RA), DBA/1J mice are used as the RA model of collagen-induced arthritis (CIA). Phloretin, a flavonoid compound extracted from* Prunus mandshurica*, has been found to exhibit anti-inflammatory activity, making it a potential candidate for treatment of RA. The objective of this study was to evaluate the therapeutic effects of phloretin on CIA mice. CIA mice were dosed daily with phloretin at either 50 or 100 mg/kg among two treatment groups. CIA treated mice showed mitigation of clinical symptoms of RA in addition to reduced inflammation of hind-limbs compared to mice who did not receive phloretin. Histological analysis showed that phloretin suppressed the severity of RA and effectively mitigated joint inflammation and cartilage- and bone-destruction via reducing proinflammatory cytokine productions (TNF-*α*, IL-6, IL-1*β*, and IL-17). This was at least partially mediated by causing inadequate splenocyte activation and proliferation. Moreover, phloretin-treated CIA mice showed decreased oxidative stress and diminished levels of malondialdehyde (MDA) and hydrogen peroxide (H_2_O_2_) in paw tissues as well as reduced productivity of anti-collagen antibodies in serum. We have concluded that phloretin could be a potent and effective antiarthritis agent, demonstrating anti-inflammatory, antioxidative, and immunomodulatory effects in CIA mice.

## 1. Introduction

Rheumatoid arthritis (RA) is a chronic and systemic autoimmune disease. Collagen-induced arthritis (CIA) in a murine model has been widely used to investigate the pathogenesis and the progression of RA and to explore possible antiarthritis agents [1]. The pathogenesis of RA is unclear, but uncontrolled immune response is considered a major pathogenic factor, spawning aberrant production of inflammatory cytokines such as TNF-*α*, IL-1*β*, IL-6, and IL-17 [2–5] and autoantibodies against citrullinated peptides [6, 7]. Along with abnormal immune responses, the oxidative stress caused by reactive oxidative species (ROS) might play a role in RA pathogenesis, because ROS can degrade isolated proteoglycans, and hypochlorous acid (HOCl) can fragment collagen and inhibit cartilage proteoglycan synthesis [8]. In addition, serum in RA patients showed elevated ROS levels [9]. As a result, antioxidant agents are considered to be potential treatments for this disease.

Current treatment of RA inflammation falls into two main categories: nonsteroidal anti-inflammatory drugs (NSAIDs) and cytokine receptor inhibitors, such as Tocilizumab (anti-IL-6 receptor) [10] and Amgen (anti-IL-17) [11]. However, side effects including gastritis, ulceration, or gastric hemorrhage by NSAIDs [12, 13] and elevation of cholesterol by Tocilizumab [14] are issues when using these drugs on a long-term basis. Therefore, an alternative RA therapy is required for long-term treatments.

A natural flavonoid, phloretin is extracted from* Prunus mandshurica* and has demonstrated anti-inflammatory and immunomodulatory activities from previous studies [15–17]. For example, phloretin has been found to prevent T cell activation by downregulating expression of CD69 and CD25 and reduce activated macrophage-induced inflammation by suppressing the nuclear translocation of NF-*κ*B and phosphorylation in the MAPK signaling pathway. Furthermore, phloretin can implement its antioxidative function to further reduce the inflammation [18]. With its anti-inflammatory and antioxidative properties, phloretin could be a good candidate as an RA remedy agent. Therefore, the aim of this study was to investigate the antiarthritic activities and immunomodulation of phloretin in CIA mice and evaluate its possibility for treatment of RA.

## 2. Material and Methods

### 2.1. Animals

8-week-old male DBA/1J mice (20–22 g weight) from Jackson Laboratory (Bar Harbor, Maine, USA) were housed under specific-pathogen-free (SPF) condition. All animals were treated in accordance with the Institutional Animal Care and Use Committee (IACUC) of National Chung Hsing University (NCHU), and the study protocols were approved by the Committee on Animal Research and Care in NCHU.

### 2.2. Collagen-Induced Arthritis (CIA)

CIA induction was performed in accordance with protocols adapted from previous studies with slight modification [19, 20]. Briefly, bovine collagen type II (CII) (Chondrex, Inc., WA, USA), was dissolved to 2 mg/mL by 10 mM acetic acid followed by emulsification with complete Freund's adjuvant (CFA). Equal volumes of bovine CII and CFA were mixed together with heat-inactivated* Mycobacterium tuberculosis* H37Ra (250 *μ*g/mouse) (Difco laboratories Inc., MI, USA). The mixture was injected intradermally into mice (200 *μ*L/mouse) at the base of the tails. Thereafter, booster doses of CII in incomplete Freund's adjuvant (IFA) were given to mice at day 21 after primary immunization (200 *μ*L/mouse).

### 2.3. Treatment

Phloretin was purchased directly from Sigma Aldrich Co. (St. Louis, MO, USA) and dissolved in dimethyl sulfoxide and diluted in PBS for oral administration. Two phloretin-treated groups (*n* = 5 for each group) were given 50 and 100 mg/kg of phloretin orally once a day for the entire period of experimentation except naïve and CIA groups.

### 2.4. Clinical Assessment of Arthritis

Clinical arthritis was assessed two days in a week for up to 6 weeks after primary CII-immunization and arthritic scores were recorded by examiners blinded to the group conditions. Scales (0–4) of clinical symptoms used to evaluate the severity of arthritis are as follows: 0 = no evidence of erythema and swelling; 1 = erythema and mild swelling confined to the tarsals or ankle joint; 2 = erythema and mild swelling extending from the ankle to the tarsals; 3 = erythema and moderate swelling extending from ankle to metatarsal joints; and 4 = erythema and severe swelling encompass the ankle, foot and digits, or ankylosis of the limb [19].

### 2.5. Histological Analysis

For histologic examination, mice from each group were sacrificed and the hind-limbs were collected at the end of the experiments. Limbs were fixed in 10% buffered formalin and decalcified in 15% EDTA before paraffin section. Tissue slides were stained with hematoxylin and eosin (H&E) according to standard methods. Histopathological changes, such as cell infiltration, cartilage destruction, and bone erosion, were scored and defined as the previous study described [21]. In short, 0 = normal joint structure; 1 = mild changes, synovitis, and pannus front with few discrete cartilage focal erosions; 2 = moderate changes, accompanying loss of large areas of cartilage, eroding pannus front, and synovial hyperplasia with infiltrating inflammatory cells; and 3 = severe synovitis, cartilage and bone erosion, and destruction of joint architecture.

### 2.6. CII-Induced Cytokine Production Analysis

The dissected hind paw tissues were rinsed and homogenized in iced normal saline by homogenizers. The homogenates were immediately centrifuged twice at 3000 rpm for 10 minutes at 4°C to isolate supernatant for subsequent cytokine quantifications. Splenocytes from naïve or CIA mice were planted with RPMI-1640 supplemented with 10% FBS into 24-well plates (1 × 10^6^ cells/well). Supernatants were collected after 48 h culture with or without 5.0 *μ*g/mL Con A (Sigma-Aldrich St. Louis, MO, USA). Cytokine concentrations, including TNF-*α*, IL-1*β*, IL-6 (eBioscience, San Diego, CA, USA), and IL-17A (R&D Systems Inc., Minneapolis, MN, USA), were measured by standard sandwich ELISA according to the manufacturer's protocol.

### 2.7. Oxidative Markers Analysis

Malondialdehyde (MDA) levels were measured by thiobarbituric acid reactive substances (TBRAS) assay at 532 nm. A standard curve was established using 1,1,3,3-tetramethoxypropane. In parallel, hydrogen peroxide (H_2_O_2_) concentrations were quantified by using Hydrogen Peroxide Assay Kit (Biovision Inc., CA, USA), following the manufacturer's protocol. The MDA and H_2_O_2_ levels were presented in unit of nmol/g protein.

### 2.8. Identification of Anti-Collagen Antibodies in Serum

Blood was collected from the mouse hearts on the last day of the phloretin treatment and centrifuged to obtain serum. Sera were serially diluted 2-fold in Tris-buffered saline (pH 8.0) containing 1% BSA and 0.5% tween-20, transferred to 96-well plates, and left overnight at 4°C. After five washes with 0.05% tween-20 in PBS, bound IgG in the plate was detected by incubating with 1 : 5000 dilution of HRP-conjugated sheep anti-mouse IgG (Jackson Immunoresearch Laboratories, PA, USA). Each plate was washed again and developed with ABTS substrate (Roche Diagnostic Systems, CA, USA). The developing reactions were stopped with H_2_SO_4_ prior to an optical density (OD) measurement at 450 nm with an ELISA reader (Sunrise™, Tecan Inc., Switzerland).

### 2.9. Cell Proliferation Assay

Splenocytes were cultured at 4 × 10^5^ cells/well with or without CII for 40 hours. Cells were pulsed with 1 *μ*Ci/well of [^3^H]-thymidine (MP Biomedicals, Solon, OH, USA) followed by an additional 8-hour incubation. Splenocytes were then harvested and assessed for the incorporation of radioactivity.

### 2.10. Statistical Analysis

Data were presented as mean ± SD. Statistical analysis was performed using one-way ANOVA with subsequent Tukey's HSD test. Statistically significant differences between groups were considered if *p* value is less than 0.05 (*p* < 0.05).

## 3. Results

### 3.1. Inhibitory Effects of Phloretin on Collagen-Induced Arthritis (CIA)

**Figure 1:**
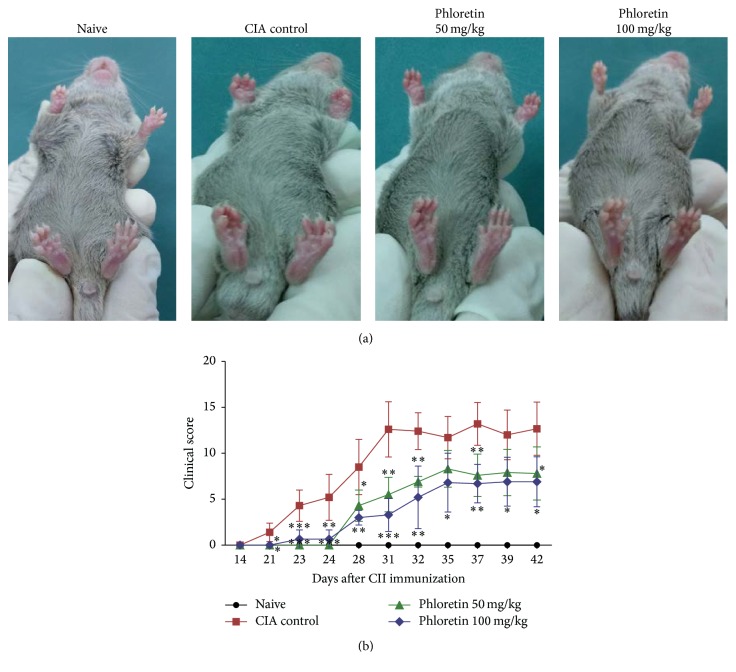
The effects of phloretin on the development and clinical of CIA. (a) Photograph type (hind paw volume). (b) Clinical scores of CIA were monitored after booster immunization. Each point on the graph represents the mean ± SD of five mice. The data presented are representatives of three independent experiments with similar results. ^**∗**^ *p* < 0.05, ^**∗∗**^ *p* < 0.01, and ^**∗∗∗**^ *p* < 0.001 versus CIA control group.

**Figure 2:**
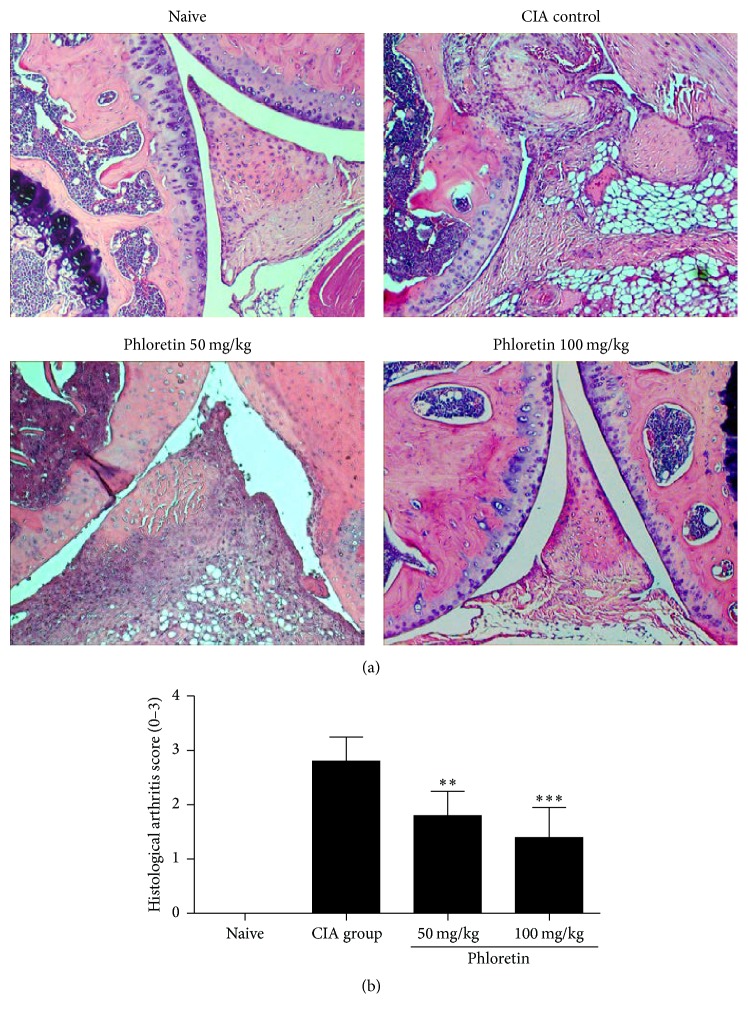
Histological analysis of the sections of knee joints on day 42. (a) Sections of knee joint sections were stained with hematoxylin and eosin. Original magnification ×100. (b) The pathogenic score was determined. Data expressed as means ± SD of five mice in each group. The data presented are representatives of three independent experiments with similar results. ^**∗∗**^ *p* < 0.01 and ^**∗∗∗**^ *p* < 0.001 versus CIA control group.

We utilized the CIA mouse model to assess the therapeutic effects of phloretin on the progression of RA. As previously mentioned, mice were dosed daily with phloretin, 50 and 100 mg/kg among two groups, and the clinical scores of RA were evaluated periodically after bovine type II collagen (CII) immunization. We found that phloretin-treated mice exhibited less severe CIA in hind-limbs (Figure 1(a)) and lower clinical scores (Figure 1(b)) in a dose-dependent manner. In addition, histological examination of mouse ankle joints showed that arthritic symptoms include extensive infiltration of inflammatory cells into articular tissues, exudation into the synovial space, synovial hyperplasia, and cartilage erosion in CIA mice but not in naïve mice. Yet, the histological scores in CIA mice were significantly lower after treating with phloretin (Figure 2).

### 3.2. Phloretin Inhibited the Production of Inflammatory Mediators in Mouse Joints

**Figure 3:**
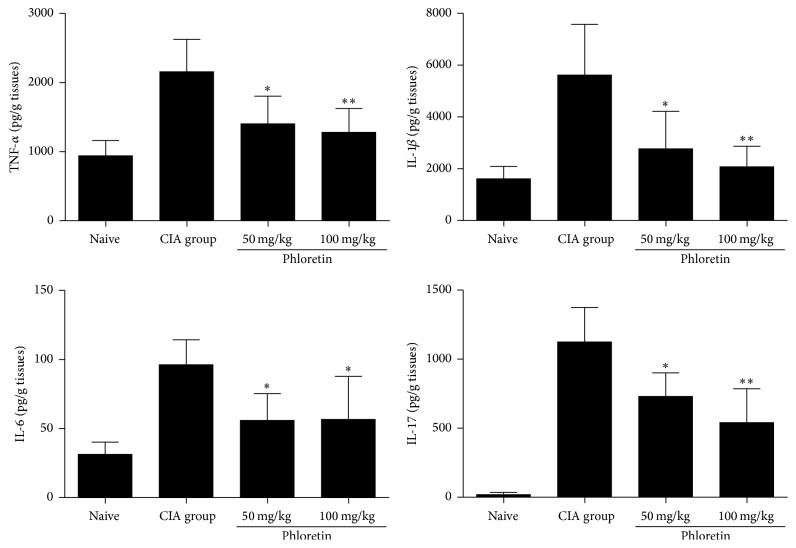
Levels of TNF*α*, IL-1*β*, IL-6, and IL-17A in hind paw homogenates. These inflammatory cytokines were measured by ELISA. The results represent the means ± SD of triplicate determinations from five mice/group. The data presented are representatives of three independent experiments with similar results. ^**∗**^ *p* < 0.05 and ^**∗∗**^ *p* < 0.01 versus CIA control group.

Since the overproduction of proinflammatory cytokines is one of essential pathological indications of RA, we investigated whether phloretin could affect the production of proinflammatory cytokines. Mice were sacrificed, the hind-limbs were removed and homogenized at the end of the experiment (day 42), and the levels of proinflammatory cytokines (TNF-*α*, IL-1*β*, and IL-6) and IL-17 were determined by sandwich ELISA. As shown in Figure 3, the levels of proinflammatory cytokines, including Th17-associated cytokine, IL-17, found in the joint of CIA mice after phloretin treatment (50 and 100 mg/kg) were significantly reduced.

### 3.3. Phloretin Suppressed the Proinflammatory Cytokine Production from Activated Splenocytes

**Figure 4:**
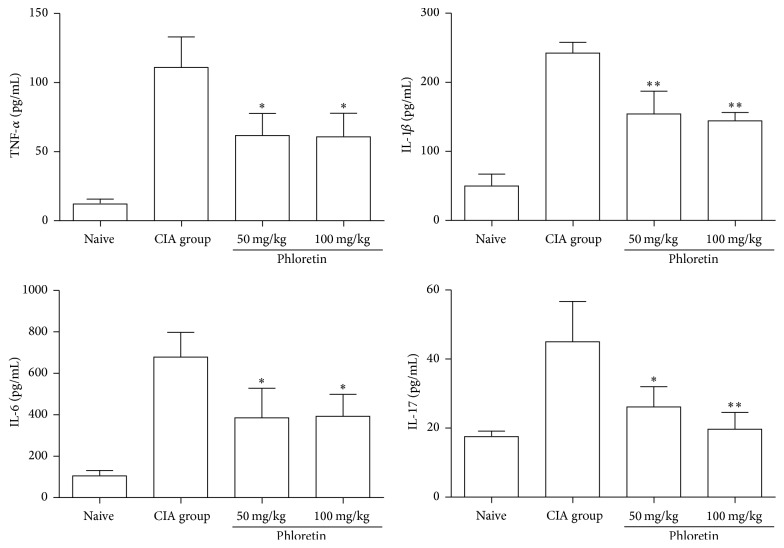
Effect of phloretin on Con A-induced inflammatory cytokine production by splenocytes* in vitro*. Splenocytes from different groups of mice were cultured for 48 h with 50 *μ*g/mL Con A. The supernatants were collected, and TNF-*α*, IL-1*β*, IL-6, and IL-17A levels were determined by ELISA. The results represent the means ± SD of triplicate determinations from five mice/group. The data presented are representatives of three independent experiments with similar results. ^**∗**^ *p* < 0.05 and ^**∗∗**^ *p* < 0.01 versus CIA control group.

Next, we studied the effects of phloretin on the Con A-activated splenocytes in CIA mice. Equivalent cell numbers of splenocytes from different groups of mice were cultured with Con A and supernatants were collected for cytokine quantification. We discovered that the Con A-induced proinflammatory cytokines produced from murine splenocytes, (TNF-*α*, IL-1*β*, IL-6, and IL-17) were significantly decreased in phloretin-treated mice compared to the CIA group (Figure 4). Both results in Figures 3 and 4 suggested that the antiarthritic activities of phloretin could be achieved via inhibiting proinflammatory cytokines in collagen-induced arthritis mouse model.

### 3.4. Phloretin Downregulated Oxidative Stress in CIA Mice

**Figure 5:**
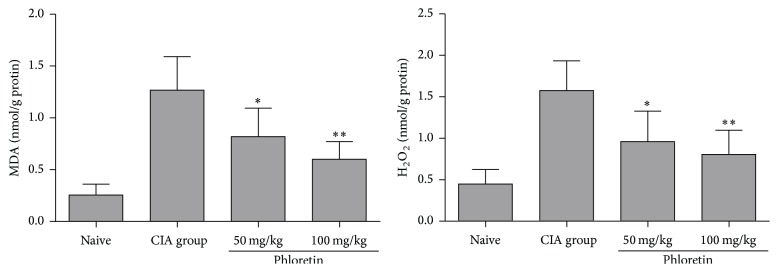
Levels of MDA and H_2_O_2_ in hind paw homogenates. These oxidative stress markers were measured by thiobarbituric acid reactive substances (TBRAS) assay and Hydrogen Peroxide Assay Kit. The results represent the means ± SD of triplicate determinations from five mice/group. The data presented are representatives of three independent experiments with similar results. ^**∗**^ *p* < 0.05 and ^**∗∗**^ *p* < 0.01 versus CIA control group.

Previous studies have shown that oxidative stress, including the production of malondialdehyde (MDA) and hydrogen peroxide (H_2_O_2_), contributes to the severity of RA [22, 23]. We measured the levels of MDA and H_2_O_2_ in CIA mice with or without phloretin treatments (Naïve and CIA groups). The results not only confirmed that the concentrations of MDA and H_2_O_2_ were higher in CIA mice than those in naïve control but also showed that the phloretin alleviated the oxidative status in CIA mice in a dose-dependent manner (Figure 5). This suggests phloretin could relieve arthritis via multiple activities.

### 3.5. Phloretin Reduced the Production of CII-Specific Antibody and the Activation of Collagen-Induced Cell Proliferation

**Figure 6:**
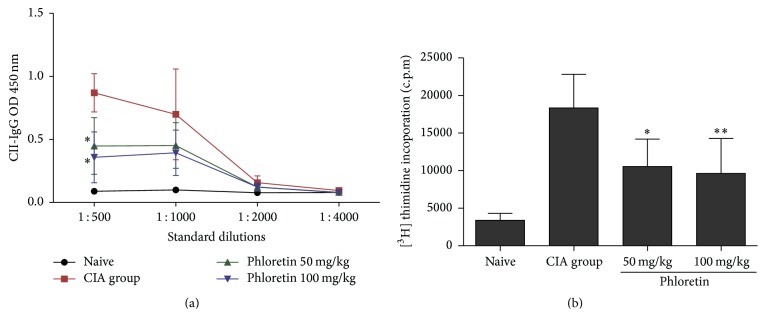
Effects of phloretin on anti-CII IgG and CII-specific cell proliferation. (a) Serum was obtained from each group on day 42 and CII-specific IgG levels were determined by ELISA. Each point on the graph represents the means ± SD of triplicate determinations from five mice/group. (b) Spleen cells were obtained on day 42 and cultured with CII for 48 h, and cell proliferation was measured by [^3^H]-thymidine incorporation assay. Data expressed as means ± SD of triplicate determinations from five mice/group. The data presented are representatives of three independent experiments with similar results. ^**∗**^ *p* < 0.05 and ^**∗∗**^ *p* < 0.01 versus CIA control group.

Autoantibodies to citrullinated protein and immunoglobulin G (IgG) are hallmarks in RA pathogenesis [6, 7]. Therefore, we assessed whether the phloretin could alleviate arthritis by reducing CII-specific antibodies. The result revealed that the production of anti-CII IgG antibody in CIA mice was suppressed after administrating phloretin (Figure 6(a)). Furthermore, we evaluated the CII antigen-induced splenocyte proliferation in CIA and phloretin-treated mice to better understand the inhibitory functions of phloretin on RA. As shown in Figure 6(b), the cellular proliferation of splenocytes from phloretin-treated mice was less potently stimulated by bovine CII antigen compared to cells from CIA mice (Figure 6(b)). These data indicated that phloretin could potentially inhibit the autoantibody production and prevent the inadequate cell proliferation of splenocytes.

## 4. Discussion

Phytochemicals from nature such as flavonoids have been applied to the inhibition of inflammation for centuries. For example,* Uncaria tomentosa* is traditionally used in Peru as an herb medicine to treat arthritis and bursitis [24]. Also, flavonoids derived from the bark of* Pinus maritime *were reported to scavenge free radicals and diminish LPS-induced IL-1*β* production [25]. Among these plant-based flavonoids, phloretin, the aglycon of phlorizin (also called phoridzin), is a multifaceted polyphenol found in apple trees and strawberries. Phloretin has been implicated in combating various diseases such as cardiovascular disease [26], biofilm-related infections [27], and inflammatory bowel diseases (IBDs) [28]. However, phloretin has not been investigated as a possible treatment of RA. Hence in this study, we demonstrated that phloretin can be a potential therapeutic agent for RA treatment.

Despite being recognized as anti-inflammatory compound, relatively little is known about the possible mechanisms of phloretin's alleviation of RA. One study in 2005 reported that phloretin could inhibit the tautomeric conversion of phenylpyruvate-related substrates conducted by macrophage migration inhibitory factor (MIF), a pro-inflammatory cytokine from T cells [29]. Another study has also shown that phloretin along with other compounds in apple juice extract downregulated the transcription levels of IFN-*γ*-inducible protein-10 (IP-10), NF-*κ*B, and IL-8, further repressing the induction of proinflammatory cytokines and chemokines [28]. Moreover, it is proved that phloretin can block NF-*κ*B and MAPK pathway to achieve its anti-inflammatory effect [30]. Another theory was proposed to explain the anti-inflammatory activity of phloretin. It was suggested that proinflammatory mediators induced by LPS, such as TNF-*α* and IFN-*γ*, increased the glucose flux via glucose transporter- (GLUT-) mediated uptake in human bronchial epithelial cells. Phloretin, a well-known GLUT inhibitor since 1962 [31], inhibits D-glucose uptake and alleviates the negative effects of inflammatory conditions [32].

Combination treatments with phloretin could be also a therapeutic strategy for treating RA. In fact, combination of disease-modifying antirheumatic drugs (DMARDs) as antiarthritic regimens has been used to treat RA clinically and the curative effects of multiple combination therapies are more effective than monotherapy in RA patients [33]. It is implicated that methotrexate and other anti-inflammatory drugs such as sulfasalazine or chloroquine could have synergistic anti-inflammatory properties [34, 35]. Therefore, combining phloretin and other antiarthritic remedies is an acceptable rationale.

## 5. Conclusions

In conclusion, our study clearly demonstrated that phloretin* per se* can alleviate the collagen-induced arthritis in mice by exerting its anti-inflammatory, antioxidation, and immunomodulatory properties. Consequently, these results suggest that phloretin could be considered as a potential candidate drug for RA treatment.
